# Spatial pattern of China’s rural digital economy based on subjective–Objective evaluation: Evidence from 2085 counties

**DOI:** 10.1371/journal.pone.0292249

**Published:** 2024-02-21

**Authors:** Xuran Liu, Fangfang Ma, Tongze Guo, Zhiwei Ding

**Affiliations:** 1 College of Geography and Environmental Science, Henan University, Kaifeng, Henan, China; 2 Key Research Institute of Yellow River Civilization and Sustainable Development Collaborative Innovation Center on Yellow River Civilization, Kaifeng, Henan, China; 3 Institute for Ocean Engineering, Shenzhen International Graduate School, Tsinghua University, Shenzhen, China; 4 College of Resources and Environment, University of Chinese Academy of Sciences, Beijing, China; 5 National Demonstration Center for Environment and Planning, Henan University, Kaifeng, Henan, China; Zhejiang University of Finance & Economics, CHINA

## Abstract

The rural digital economy plays an essential role in China’s industrial upgrading, transformation, and urban–rural integration. To determine the state of China’s rural digital economy, we constructed a county-level evaluation system using the subjective–objective evaluation method and calculated the digital economic levels of 2085 counties. Then, we analyzed the spatial distribution characteristics, spatial autocorrelation pattern, spatial disequilibrium degree, and spatial driving force of the rural digital economy at the county level using spatial analysis technology and a self-organizing feature mapping model. The results are as follows: 1) Compared with the real economy, the agglomeration effect of the digital economy was more obvious, and the economic gradient was more significant. Specifically, the dense high-value regions formed a continuous belt on the eastern coast from the Beijing–Tianjin area to the Pearl River Delta, opposite the dense low-value regions in the west. 2) There were significant differences in the rural digital economy within cities or provinces. Intraregional differences were not necessarily linked to the overall digital economy level because central and northeastern China presented a more balanced rural digital economy. 3) Digital network performance, e-commerce level, and economic vitality were identified as the core factors influencing the rural digital economy.

## Introduction

During the latter half of the 1990s, the digital economy emerged as a new economic activity in the US, accompanied by a surge in labor and total-factor productivity growth rates [1, 2]. At first, this new economy was identified based on two main features: ICT (information and communication technology) and e-commerce [3]. More recently, the concept of the digital economy has been enriched by the rapid development of new digital technologies, such as big data, cloud computing, artificial intelligence, and blockchain [4–7]. Today’s digital economy can be characterized as a series of economic activities that use Internet platforms and various digital technologies to improve economic efficiency and optimize the industrial structure.

The digital economy plays a crucial role in economic growth and industrial transformation [8]. In terms of economic growth, the effects of the digital economy have been seen in many countries, not only developing economies with weak digital infrastructure [9, 10] but also highly developed ones with advanced digital industries [11]. The literature has largely recognized a positive effect of the digital economy on economic growth, mainly attributable to changes in productivity or to externalities related to disseminating knowledge and innovation. Regarding industrial transformation, although some studies have shown that the difficulty of rural digital infrastructure construction could cause the digital economy to lag behind that of cities, showing a large urban–rural gap [12, 13], the digital economy has nevertheless played an important role [14, 15].

In China, digital economic activities, such as the Taobao platform and live e-commerce, play an important role in the process of delivering the effects of urbanization and informatization to rural areas. They also promote the urban–rural integration of infrastructure and public services [16–18]. Therefore, accelerating the growth of the rural digital economy is conducive to not only enhancing rural vitality but also promoting industrial upgrading and transformation, even serving as a force to strengthen urban–rural linkages [19]. Recently, with the gradual planning and deployment of new digital technologies, the digital economy has made various achievements in terms of promoting farmers’ income, expanding employment paths, and promoting urban–rural integration in central and western China [20]. Online retail sales reached 3530.3 billion at the county level in 2020, an increase of 14.02% over the previous year, and the overall level of the rural digital economy at the county level reached 39.1%, an increase of 3% over the previous year [21]. Although the county-level digital economy has performed well in terms of promoting rural revitalization—especially during COVID-19 and the global economic downturn—it still faces practical problems such as a lack of professional talent, insufficient innovation vitality, and unbalanced regional distribution [22, 23]. In this context, China has introduced various policies to support the rural digital economy, such as developing smart facilities and improving the information service supply. Furthermore, researchers have also proposed paths for supporting the construction of the rural digital economy through theoretical analyses, field research, and trend analyses [24–26].

The most basic and important work in digital economy research is to accurately evaluate the digital economy level of different research units. The related methods can be divided into two categories according to the evaluation objects. One is direct evaluation, which is based on defining the relevant industries in the digital economy and calculating the GDP of each. This method is normally used to measure large economies (e.g., a country or a province) with detailed industry-specific economic data. The US Census Bureau was the first to measure e-business using this method [27]. Most studies using this method evaluated the digital economy level by measuring two core sectors: ICT and e-commerce [28]. In such studies, ICT usually included computer software and hardware, telecommunication equipment and services, Internet-enabled devices, and other services or industries related to digital equipment [23, 29, 30]. By contrast, e-commerce mainly included all purchases and sales of goods and services that occurred over computer networks, including B2B, B2C, and P2P transactions [28, 29].

Other studies have suggested that GDP cannot capture some potential digital economic activities (e.g., consuming Internet software) or the potential cultural value of the digital economy [31, 32]. Simultaneously, it can be difficult to acquire complete economic data from industries in every research unit, especially in small-scale studies. In such situations, the evaluation of the digital economy was usually performed using certain indirect methods. Most studies constructed indicator systems to estimate the digital economy in different areas. The indicators they used were complex and varied but usually included three aspects: digital infrastructure, digital industry, and digital application [33–37]. Digital infrastructure is the basic support for and technical carrier of the digital economy. It has often been evaluated by indicators such as mobile users, Internet users, broadband access, and the number of mobile base stations. Digital industry is always included in different industries and can be roughly estimated by GDP. However, it can also be described by certain indirect indicators, such as the number of employees or companies in R&D or IT and the number of related services or amount of equipment. Digital application indicates the capacity of digital infrastructure and industry for life and production. It can be evaluated by express business volume, the number of official websites owned by a company or government, or other website data. Aside from constructing indicator systems, some studies also directly used digital economy companies’ data [38], digital economy data in companies’ reports [39], and even remote-sensing data for nighttime light [40] to measure regional digital economies.

In China, it is also difficult to conduct quantitative digital economy research, especially in rural areas. It is worth noting, however, that in today’s administrative divisions in China, counties and districts are generally the largest scale units that distinguish urban and rural areas. Therefore, the concept of the county-level digital economy is similar to that of the rural digital economy. For this study, therefore, the county level is not only an administrative division level but also represents the rural area. The main themes of research on China’s county-level digital economies include proposing strategies and policies and interpreting theoretical connotations [41]. For example, some studies have analyzed strategies or paths for special counties [42, 43]. In those studies, digital infrastructure construction, e-commerce economy, and digital finance were regarded as important driving forces for rural digital economy development. Moreover, industrial digitization and digital technology enterprises have been regarded as the advanced characteristics and future trends of rural digital economy development. Although the concepts and development paths of the rural digital economy have been clear enough, evaluation remains difficult. Most evaluations focus on the city or provincial levels [40, 44]. The indicator systems are more concerned with high-end digital economy industries and the deep application of the digital economy in large-scale research. However, such features are weak in rural digital economies and are difficult to evaluate on a small scale. Therefore, the construction of the rural digital economy should be reconsidered. Recently, Zhu et al. used the evolution of the point-of-interest (POI) data of digital enterprises to reflect spatial changes in industries and cities [38]. This showed that POI data could be a useful tool for reflecting the activities of the digital economy at a small scale. It is also worth mentioning that some researchers have captured the county-level digital economy in terms of Internet infrastructure, rural digitalization degree, financial application of digital technology, and constructed evaluation indicators [22, 45, 46]. Yet, all-around, multilevel systems that evaluate the county-level digital economy remain rare.

Digital economy development has become an important force for global economic and social transformation. In this process, China’s digital economy has developed particularly rapidly. From information dissemination to e-commerce, from network services to intelligent decision-making, the application modes of the digital economy are becoming increasingly diverse. However, China has a huge land area and a large population, resulting in significant regional and urban–rural differences. Therefore, policymaking and planning must be undertaken separately for different regions. This is reflected in the government’s formulation of different regional development strategies, such as the Yangtze River Economic Belt [47–49], the Yellow River Basin Ecological Protection and High-Quality Development Strategy [50], and the Beijing–Tianjin–Hebei Integration Strategy [51]. Similarly, research should also consider these spatial differences and regional development strategies and use geographic analysis to identify the differences and provide suggestions for adapting to local conditions. This makes the related research in China very different from the situation in other countries. At the same time, smaller-scale research can better reflect spatial differences. In this context, the present study aimed to grasp the level of China’s rural digital economy based on a geographical perspective and tried to answer the following questions: 1) How can we choose reasonable indicators to construct an indicator system and determine the spatial status of China’s rural digital economy at a small scale? 2) What are the development types of the digital economy in different regions, and how should it be transformed and developed in the future? To address these questions, we first collected different kinds of data—socioeconomic data, POI data, and relevant digital indexes—to construct the research database and indicator system. Compared with traditional digital economy evaluation, POI data can more accurately reflect the vitality of the rural digital economy, and using a third-party digital index makes our research more objective. After acquiring the rural digital economy level of each county, we analyzed the grade characteristics and spatial characteristics of county-level rural digital economies using different methods: the natural breaks classification method, Exploratory Spatial Data Analysis (ESDA), GeoDetector, and the coefficient of variation. Then, through a combination of qualitative and quantitative methods, we reinterpreted the factors affecting the development of the rural digital economy. Finally, to process this large amount of county-level data and identify the main laws of the development types of the rural digital economy, we used self-organizing feature mapping (SOFM) to classify and optimize the path for each type. Our findings can support the sustainable development of China’s digital economy and the urban–rural integration of the virtual and real economies. This research can also offer guidance for digital economy development and industrial transformation in other countries.

## Indicator system, research methods, and data sources

### Indicator system

The digital economy is a new economic form based on modern information networks, and it takes data resources as its key elements [27, 30, 31]. Moreover, the digital economy is driven by the integrated application of ICT and the digital transformation of all elements [52, 53]. It has rich connotations and thus requires multidimensional evaluation. At present, evaluations of the digital economy are mainly carried out in terms of the industrial environment, industrial efficiency, and application state at the city level or province level [33, 34]. With the online transformation of the real economy and the emergence of China’s e-commerce industry, evaluating the county-level digital economy depends more on the improvement of the digital industry environment, the guarantee of digital financial investment, and the stimulation of digital economy vitality, as well as the related Internet and e-commerce effects. Thus, we constructed an indicator system based on those aspects. However, evaluating the county-level digital economy is difficult because of incomplete economic data. Therefore, we also referred to some relevant indicators and POI data to measure county-level digital economies. Then, we constructed a comprehensive indicator system based on the industrial environment, digital index, development vitality, Internet effect, and e-commerce level (Table 1). We chose 2019 as the measurement year based on the recency and availability of data.

**Table 1:** Indicator system for county-level digital economies in China.

Primary indicators	Secondary indicators	Subjective weights	Objective weights	Comprehensive weights
Industrial environment *C1*	GDP per capita *P1*	0.042	0.039	0.047
	Share of the tertiary industry in GDP *P2*	0.052	0.007	0.022
	Urbanization rate *P3*	0.034	0.011	0.023
Digital index *C2*	Digital financial inclusion index *P4*	0.071	0.012	0.034
	Digital village index *P5*	0.088	0.008	0.031
Development vitality *C3*	Number of telecom business halls per 10,000 people *P6*	0.085	0.018	0.045
	Number of Internet technology companies per 10,000 people *P7*	0.104	0.099	0.119
	Number of digital electronics stores per 10,000 people *P8*	0.068	0.028	0.051
	Number of shared equipment stations per 10,000 people *P9*	0.068	0.132	0.112
Internet effect *C4*	Baidu search results number *P10*	0.042	0.047	0.052
	Baidu index: search index *P11*	0.052	0.051	0.061
	Baidu index: information index *P12*	0.064	0.386	0.185
E-commerce level *C5*	E-commerce development index *P13*	0.088	0.028	0.059
	Internet business index *P14*	0.071	0.098	0.098
	Online shopping index *P15*	0.071	0.036	0.059

Subjective–objective evaluation integrates the advantages of subjective and objective evaluation. It can not only avoid subjective bias by experts but also offer the support of objective results [54]. We used the entropy method and analytic hierarchy process (AHP) to calculate subjective and objective and weights, respectively.

The steps for calculating subjective weights are as follows:

Calculate the proportion *p*_*ij*_ of the *i*th research unit under the *j*th indicator in this indicator:

$$p_{ij}=\frac{x_{ij}}{\sum_{i=1}^{n}x_{ij}}(i=1,2,\ldots ,n;j=1,2,\ldots ,m),$$ (1)

where *x*_*ij*_ is the score of the *i*th research unit in the *j*th indicator, *n* is the total number of research units, and *m* is the total number of research indicators.

Calculate the entropy value *e*_*j*_ of the *j*th index:

$$e_{j}=-k\sum_{i=1}^{n}p_{ij}ln(p_{ij}),k>0;k=1/ln(n);e_{j}\geq 0,$$ (2)

where *k* is the reciprocal of the logarithm of *n*.

Calculate the difference coefficient *g*_*j*_ of the *j*th indicator:

$$g_{j}=\frac{1-e_{j}}{m-E_{e}},E_{e}=\sum_{j=1}^{m}e_{j},0\leq g_{i}\leq 1;\sum_{j=1}^{m}g_{j}=1,$$ (3)

where *E*_*e*_ is the sum of entropy of each index.

Calculate the weight *w*_*j*_:

$$w_{j}=\frac{g_{j}}{\sum_{j=1}^{m}g_{j}}(1\leq j\leq m),$$ (4)

The steps for calculating objective weights are as follows:

1. Establish a hierarchical structure system for importance scoring by experts.
2. Find 10 experts in the field of the digital economy to score each indicator and then calculate the average score given by each expert.
3. Round the average of expert scores to integer digits to construct a judgment matrix.
4. Enter the data into AHP software for weight calculation.
5. Adjust the weight matrix and perform the CR value test. It is worth noting that the calculation can only be stopped when all CR values are less than 0.1, because this means the consistency test has been passed.

Table 2 shows the example results.

**Table 2:** Indicator weights; test values of the industrial environment system.

Secondary indicators	P1	P2	P3	Weights
**P1**	1.00	0.80	1.25	0.042
**P2**	1.25	1.00	1.50	0.052
**P3**	0.80	0.67	1.00	0.034

Note: CI = 0, RI = 0.58, CR = 0 < 0.1

Based on the subjective and objective weights, the geometric mean method was used to calculate the comprehensive weights. The formula is as follows:

$$w_{j}=\frac{\sqrt{w_{jo}w_{js}}}{\sum_{j=1}^{m}\sqrt{w_{jo}w_{js}}}(1\leq j\leq m),$$ (5)

where *w*_*j*_ is the comprehensive weight of the *jth* indicator, *w*_*jo*_ is the objective weight of the *jth* indicator, and *w*_*js*_ is the subjective weight of the *jth* indicator. Table 1 shows the results.

### Research methods

**Fig 1:**
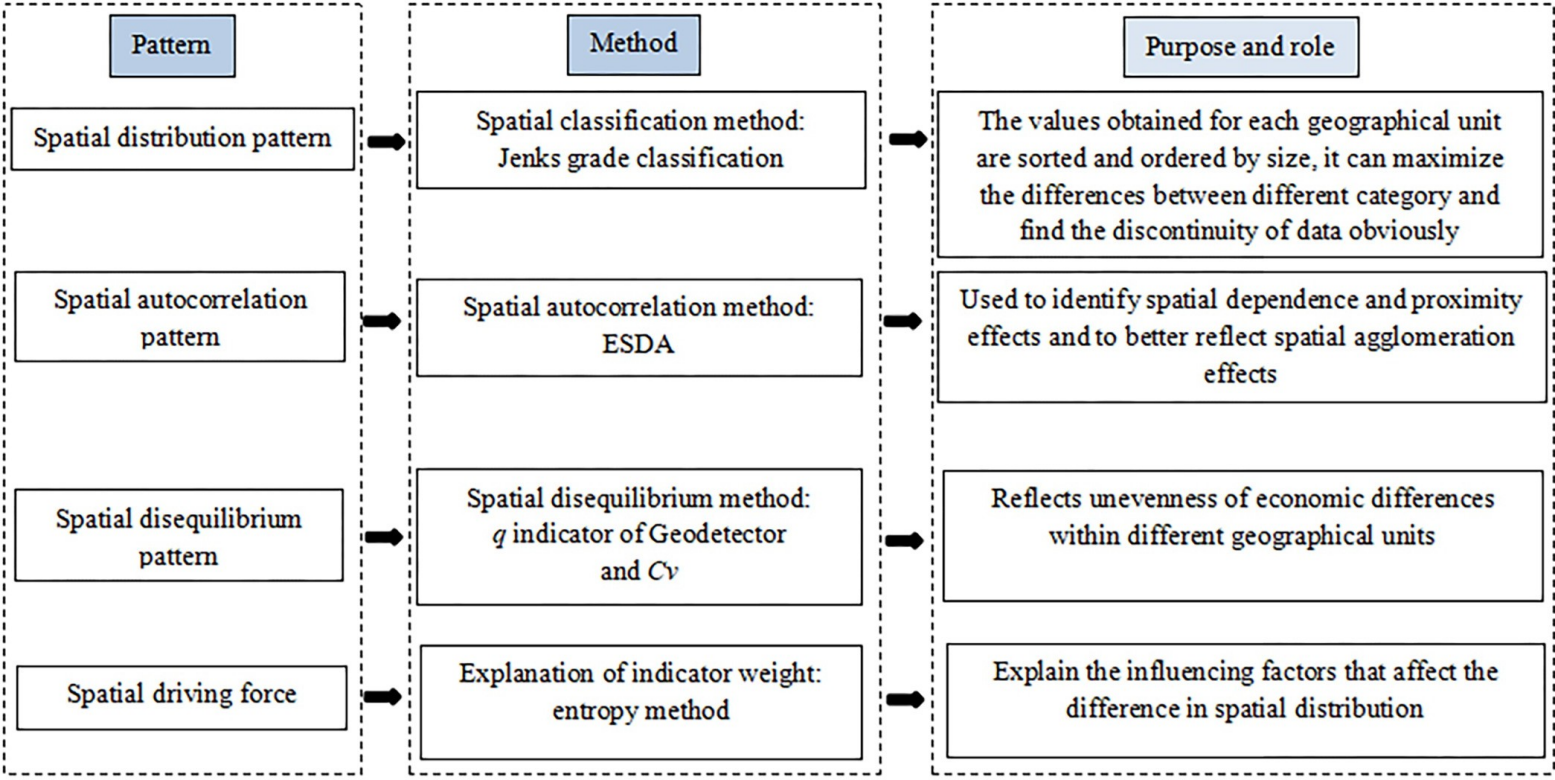
Interaction of the research method.

To study the county-level spatial characteristics of the rural digital economy, we used four methods to analyze spatial patterns: spatial distribution, spatial autocorrelation, spatial disequilibrium, and spatial driving force. Fig 1 shows the interaction of the research method.

#### Spatial classification method

Natural break classification is a statistical method of sorting and classification according to the law of mathematical distribution. It can maximize the differences between different categories and find discontinuities in data [55]. Thus, we used this method to classify the scores of each unit into five categories to map the spatial differences in China’s digital economy.

#### Spatial autocorrelation method

ESDA is a method used to explore spatial autocorrelations among research units. It can effectively identify spatial dependencies and proximity effects. Therefore, this method is used for global spatial agglomeration state identification and local regional relationship analysis. It generally includes two indexes: global spatial autocorrelation and local spatial autocorrelation. Global spatial autocorrelation is expressed by Moran’s *I* index, ranging from −1 to 1. It is mainly used to identify the overall spatial clustering state. It indicates negative spatial autocorrelation, no spatial autocorrelation, or positive autocorrelation if Moran’s *I* index is greater than 0, equal to 0, or less than 0, respectively. The formulation is as follows [56]:

$$Moran’s\;I=\frac{\sum_{i}^{n}\sum_{j}^{n}w_{ij}(x_{i}-\bar{x})(x_{j}-\bar{x})}{s^{2}\sum_{i}^{n}\sum_{j}^{n}w_{ij}},$$ (6)

where *w*_*ij*_ is the spatial weight between units *i* and *j*; *x*_*i*_ and *x*_*j*_ are the scores of unit *i* and *j*, respectively; $\bar{x}$ is the average score of all units; and *s*^2^ is the variance of the scores for all units.

The local index of spatial autocorrelation (LISA) is an index for measuring whether there are similar or different attribute clusters. It is of great significance for identifying the relationship between a county and its surrounding counties. In general, the results can be divided into five groups: significant high–high (HH), significant low–low (LL), significant high–low (HL), significant low–high (LH), and no significance. In the HH group, high-level units are surrounded by high-level units, which produces a positive aggregation-promoting effect. In the LL group, low-level units are surrounded by low-level units, which produces a negative effect of low-value agglomeration. In the HL group, high-level units are surrounded by low-level units, which produces a certain polarization effect. In the LH group, low-level units are surrounded by high-level units, which produces a localized collapse effect. No significance means there is no significant spatial interaction between the research unit and its surrounding units.

#### Spatial disequilibrium degree method

GeoDetector is a new statistical method for detecting spatial heterogeneity and revealing the driving factors. In this method, the *q* indicator is used to detect the global spatial disequilibrium degree. We used this indicator to explore whether rural digital economies had significant internal differences in different cities and provinces. The formula for the *q* indicator is as follows [57]:

$$q=1-\frac{\sum_{h=1}^{L}N_{h}\sigma_{h}^{2}}{N\sigma^{2}},$$ (7)

where *h* = 1,…, *L* represent the division at the provincial or city level; *N*_*h*_ and *N* are the number of county-level units in layer *h* and the whole country, respectively; and *σ*_*h*_^2^ and *σ*^2^ represent the score variance in layer *h* and the whole country, respectively.

The local internal disequilibrium degree of each province can be measured by *C*_*V*_; its calculation formula is as follows [58]:

$$c_{v}=\frac{\sqrt{\sum \frac{{\left(S_{i}-\bar{S}\right)}^{2}}{n}}}{\bar{S}},$$ (8)

where *C*_*v*_ is the coefficient of variation of the score in each province, *S*_*i*_ is the score of the *ith* county in each province, *n* is the total number of county units in each province, and $\bar{S}$ is the average value of the county-level score of each province.

#### Spatial driving force method

Limited by available county-level data, it is difficult to apply certain quantitative analysis methods, such as correlation analysis and regression analysis, to analyze the influencing factors. However, the weight of each indicator calculated by the entropy method can effectively reflect the indicator’s contribution to the overall level of the digital economy. This provides a new pathway for quantitative interpretation. Therefore, we combined the entropy weight and spatial differentiation of each indicator to comprehensively analyze the factors affecting spatial differentiation in rural digital economies.

### Data sources

Socioeconomic data mainly came from the China County Statistical Yearbooks (county and city volumes) and provincial statistical yearbooks for 2020; some data came from counties’ and cities’ statistical yearbooks in each province in 2020. The digital village index was acquired from the Ali Research Institute [46]. The digital financial inclusion index came from the China Digital Financial Inclusion Index (https://tech.antfin.com/research/data). Development vitality data were obtained from various POI data retrieved using Gaode API. The Internet effect data were mainly obtained from Baidu. E-commerce-level data came from the Ali Research Institute. In addition, the geographical base map, including 2,085 research units, came from the standard map website of the Ministry of Natural Resources of the People’s Republic of China.

## Results

### Spatial distribution pattern

**Fig 2:**
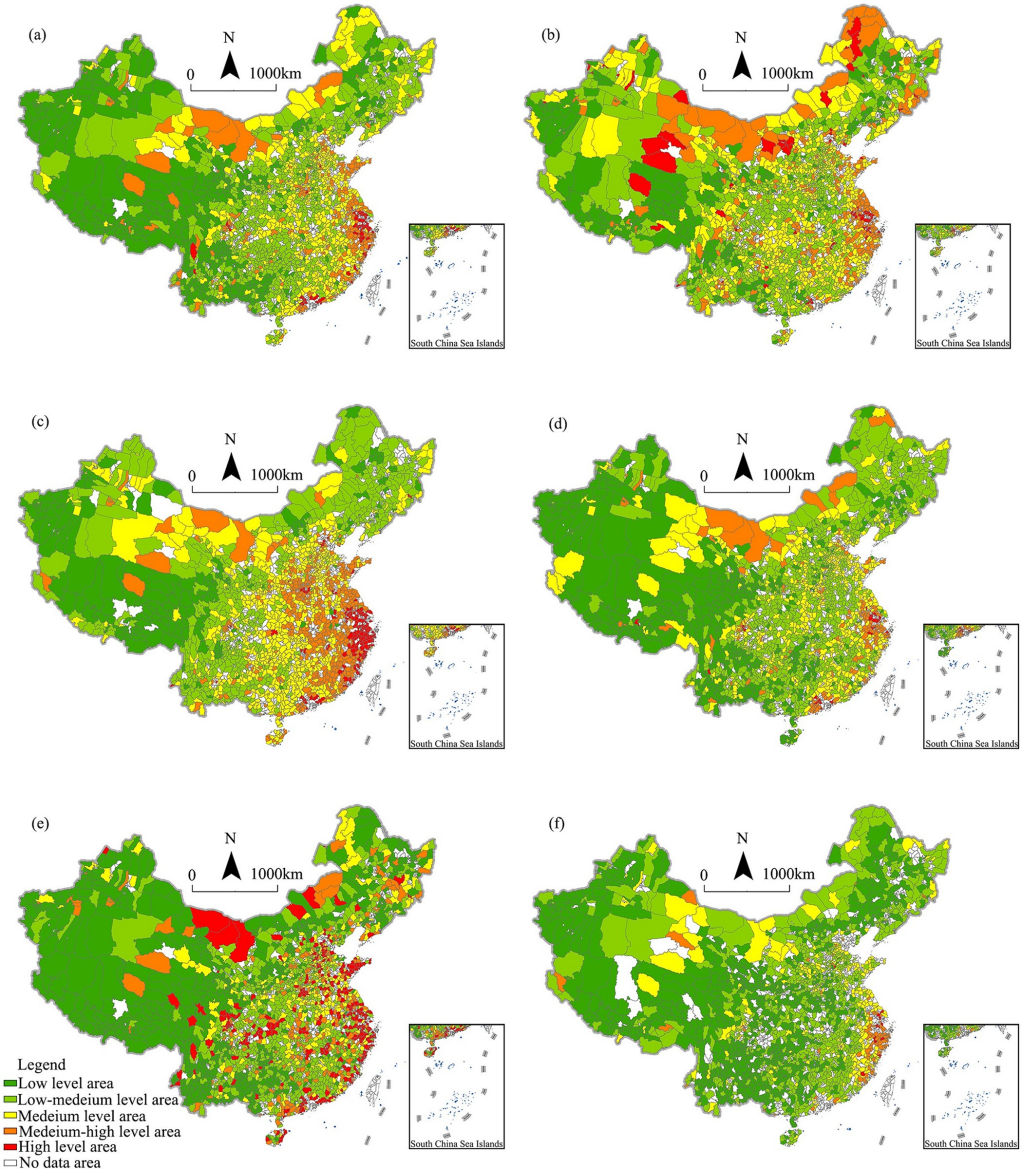
Spatial distribution pattern of China’s rural digital economy. (a) Overall level, (b) industrial environment, (c) digital index, (d) development vitality, (e) Internet effect, (f) e-commerce level.

Based on the digital economy scores, we used natural break classification to divide the scores into five categories and then map them to reflect the spatial distribution of the scores in each research unit. Fig 2 shows the results.

Fig 2(A) shows that high- and medium-high-level areas formed several agglomeration groups in the eastern coastal area, especially in the Beijing–Tianjin–Hebei Region, Yangtze River Delta, and Pearl River Delta. However, their scope and internal connectivity differed significantly. In general, the Yangtze River Delta had the highest rural digital economy level and local ties, especially the counties (or districts) in Zhejiang and Shanghai. Compared with the Beijing–Tianjin–Hebei region and Pearl River Delta, the Yangtze River Delta had a better regional collaborative effect in the rural digital economy, even showing a leading role in China.

The number of high-level and medium-high-level areas was not high, accounting for only 14.5%. They formed several agglomeration groups in the eastern coastal area, especially in the Beijing–Tianjin–Hebei region, Yangtze River Delta, and Pearl River Delta. This is mainly because these counties are close to metropolis areas and therefore have good industrial environments and some high-quality modern service industries (e.g., Pidu in Chengdu). Also, some counties are relatively independent and mainly rely on characteristic industries, such as Shangri-La in Yunnan. The medium- and low-medium-level areas were the most widely distributed, accounting for 63.5%. Although the county scores for those two levels were similar, the distribution patterns were markedly different. Specifically, most of the medium-level regions were concentrated in eastern and central China and were distributed around the medium-high-level and high-level areas. Therefore, these medium-level regions can absorb the achievements and experiences of the medium-high-level and high-level areas. By contrast, the low-medium-level areas were concentrated in southwestern, northwestern, and northeastern China. This is mainly because the low-medium-level areas had weak industrial foundations and low economic growth rates, resulting in underdeveloped modern service industries and digital economies. The low-level-areas were mainly distributed in the underdeveloped areas in western China, accounting for 22.0%. This distribution is mainly attributable to the fact that the rural digital economy is limited in those regions by a low speed of informatization, poor industrial transformation, and insufficient socioeconomic development.

Regarding subsystem indicators, the spatial differentiation patterns of the digital index, development vitality, and e-commerce level were similar to the overall development level (Fig 2(C), 2(D) and 2(F)). Further analysis showed that high-level-areas in these three subsystems were mainly distributed in the Yangtze River Delta and the coastal high-level socioeconomic development region. This not only reflects the advantages of these regions in terms of digital infrastructure, residential digital application, and e-commerce economy but also confirms the leading role of these regions in the upgrading of county-level economies. However, to be specific, the quantity and distribution of the high-digital-index areas were well above the overall level. This also indicates that the county-level digital economy has a good foundation for developing primary applications in eastern China, especially in the Yangtze River Delta. However, in terms of development vitality and e-commerce, the number of high- and medium-high-level areas and the scope of spatial agglomeration areas were below the overall level. This indicates that the digital economy in most central and western counties was still in the initial stage of development. Infrastructure construction and digital-inclusive finance guidance are the typical characteristics in these regions, while industrial development and e-commerce industry lack vitality. Unlike other indicators, in terms of the industrial environment and network effect (Fig 2(B) and 2(E)), there were more high-level and medium-high-level counties distributed in central and western China than the overall level, owing to online marketing, Internet branding, and specialized network business industries.

### Spatial autocorrelation pattern

To investigate the spatial autocorrelation characteristics of the rural digital economy, we calculated the global and local Moran’s indexes. Table 3 and Fig 2 show the results. We can see that the Moran’s index of different scales was greater than 0.3, and the *p*-value was less than 0.01, whether at the county, city, or provincial level. These results indicate obvious spatial agglomeration in China’s rural digital economy.

**Table 3:** Global spatial autocorrelation results for digital economy development levels at different scales.

Indicator	County level	City level	Province level
**Moran’s *I***	0.38	0.49	0.35
***p*-value**	0.00	0.00	0.00

**Fig 3:**
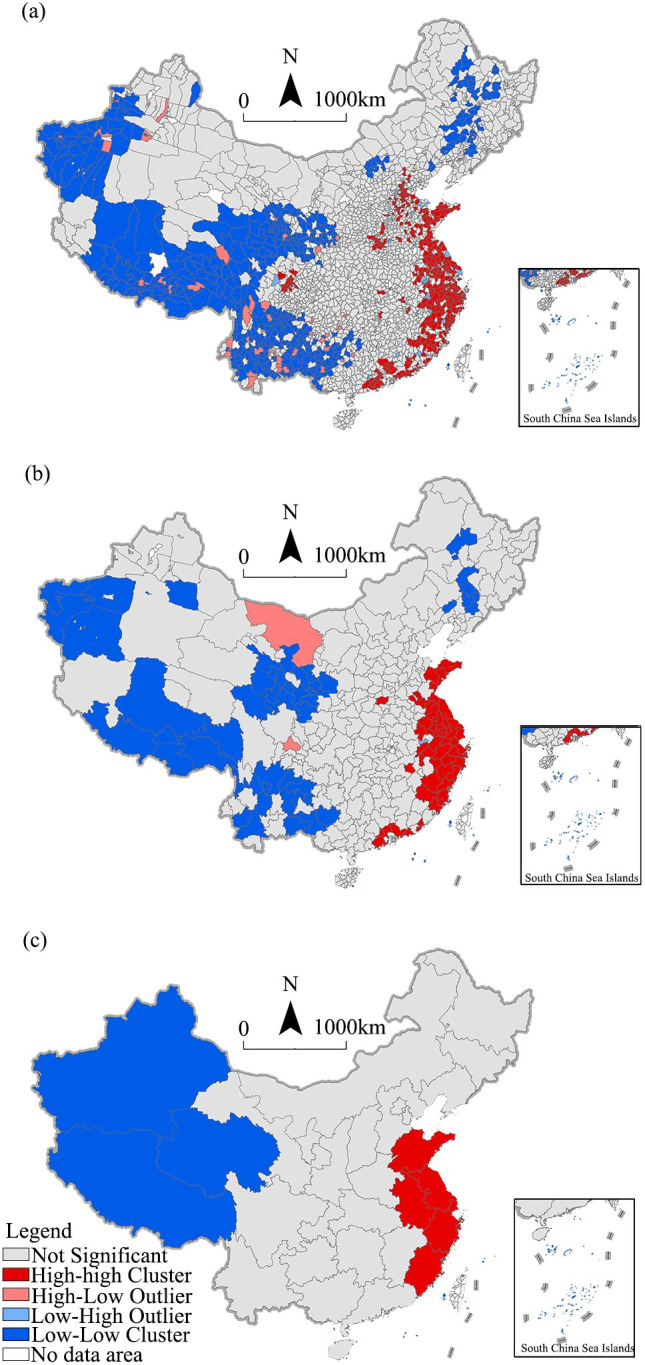
LISA map of the digital economy at different levels. (a) County level, (b) city level (average of the county level), (c) province level (average of the county level).

Specifically, at the county level (Fig 3(A)), the significant HH counties formed a strip shape from the Beijing–Tianjin region to the Yangtze River Delta and the Pearl River Delta in the coastal region. This is mainly because of the high degree of economic interaction and infrastructure sharing in these regions. The significant LL counties were mainly distributed in western China, especially in the Qinghai–Tibet Plateau and the border areas of northwestern China. In these regions, the disadvantaged geographical environment and industrial basis restrict the rural digital economy. Compared with the county level, the agglomeration effect and spatial heterogeneity of the rural digital economy cannot be well identified at city level and provincial level. At the city level, the significant HH areas in the Beijing–Tianjin area disappeared (Fig 3(B)) and had shrunk in the Pearl River Delta area. However, the scope of significant HH counties was still large in the Yangtze River Delta and its adjacent regions. Although the distribution pattern was similar to that of the county level, its scope was smaller than that of the county level. This is mainly because of the insufficient digital transformation and upgrading of the Beijing–Tianjin–Hebei and Pearl River Delta regions. Also, the divergence of urban–rural informatization levels is significant, and e-commerce vitality is not high in these regions. At the provincial level (Fig 3(C)), the significant HH areas were still agglomerated in eastern China. This further reflects the leading level and dominant position of the Yangtze River Delta urban agglomeration. The significant LL areas at the provincial level, including Xinjiang, Qinghai, and Tibet, were spatially opposite to the significant HH areas, reflecting the economic differences between western and eastern China.

### Spatial disequilibrium pattern

The *q*-value of geographic detectors and *Cv* were used to identify internal differences between rural digital economies at the city and provincial levels. Table 4 shows the *q*-value results. We can see that the city-level value (0.6706, *p* < 0.01) is generally larger than the provincial-level value (0.4221, *p* < 0.01), meaning the digital economy at the city level could be more appropriate for expressing the county level to some extent. From the comparison of the subsystem level, the *q*-values of the digital index and e-commerce level were higher, indicating that rural digitization, digital-inclusive finance, and e-commerce are the general concerns of county governments. On the contrary, the *q*-values of industrial environment, development vitality, and Internet effect were lower. They are the main reasons for the internal differentiation of the rural digital economy in different cities or provinces.

**Table 4:** The *q*-values of digital economy development levels at provincial and city scales.

Indicator	Overall level	Industrial environment	Digital index	Development vitality	Internet effect	E-commerce level
***q*-value at city level**	0.6706	0.5177	0.8090	0.6079	0.4526	0.6613
***p*-value**	0.00	0.00	0.00	0.00	0.00	0.00
***q*-value at province level**	0.4221	0.2388	0.6259	0.2973	0.2491	0.4545
***p*-value**	0.00	0.00	0.00	0.00	0.00	0.00

We used the calculation results for *Cv* to classify the degree of internal differences in each province. Table 5 shows the results. Specifically, the high-disparity areas were Qinghai and Tibet. In these provinces, the industrial environment varies markedly from county to county. Thus, there was no stable gradient difference in the digital economic level in western China. However, the medium-high-disparity areas included not only low- and low-medium-level areas such as Xinjiang, Sichuan, and Yunnan but also high-level areas such as Shanghai and Guangdong in eastern China. The reason is that in high-level areas, rural digital economy development is quite different in highly urbanized areas and less urbanized areas. For example, in Guangdong, the development level of the digital economy in the Pearl River Delta region is significantly higher than in other regions. This difference in urbanization level has caused significant differences in rural information infrastructure, digital economy sharing services, and Internet application levels. The medium-disparity-degree areas include not only Zhejiang and Fujian, with high-level economies in eastern China, but also medium-level areas like Hunan and even underdeveloped areas like Gansu and Inner Mongolia. This also indicates that intraregional differences are not necessarily linked to overall digital economy levels. The low-medium- and low-disparity-degree areas are mainly distributed in provinces with low economic and informatization levels in eastern and central China. This is mainly because the digitalization process in these areas is slow, and the driving ability of high-level counties is not strong. It is also worth noting that although the internal disparity degree was not obvious in some medium-high provinces such as Hubei and Sichuan, they lack specialized and superior digital counties, and the gap between them and high-level regions such as Shanghai and Zhejiang is still high. It is important, therefore, to focus on the cultivation of high-level counties and the transformation of low-level counties in these regions, which can, in turn, promote creation and innovation.

**Table 5:** Coefficient of variation of digital economy level in each province.

Category	Coefficient of variation range	Provincial unit name
**High disparity**	0.461653–0.578022	Qinghai, Tibet
**Medium-high disparity**	0.333865–0.461652	Xinjiang, Sichuan, Yunnan, Guangdong, Shanghai
**Medium disparity**	0.241459–0.333864	Inner Mongolia, Jiangsu, Gansu, Fujian, Guangxi, Guizhou, Heilongjiang, Ningxia, Hunan, Hebei, Liaoning, Zhejiang
**Low-medium disparity**	0.199046–0.241458	Jilin, Henan, Hainan, Anhui, Shaanxi, Shanxi, Chongqing, Tianjin
**Low disparity**	0.164804–0.199045	Shandong, Hubei, Beijing, Jiangxi

### Spatial driving force

**Fig 4:**
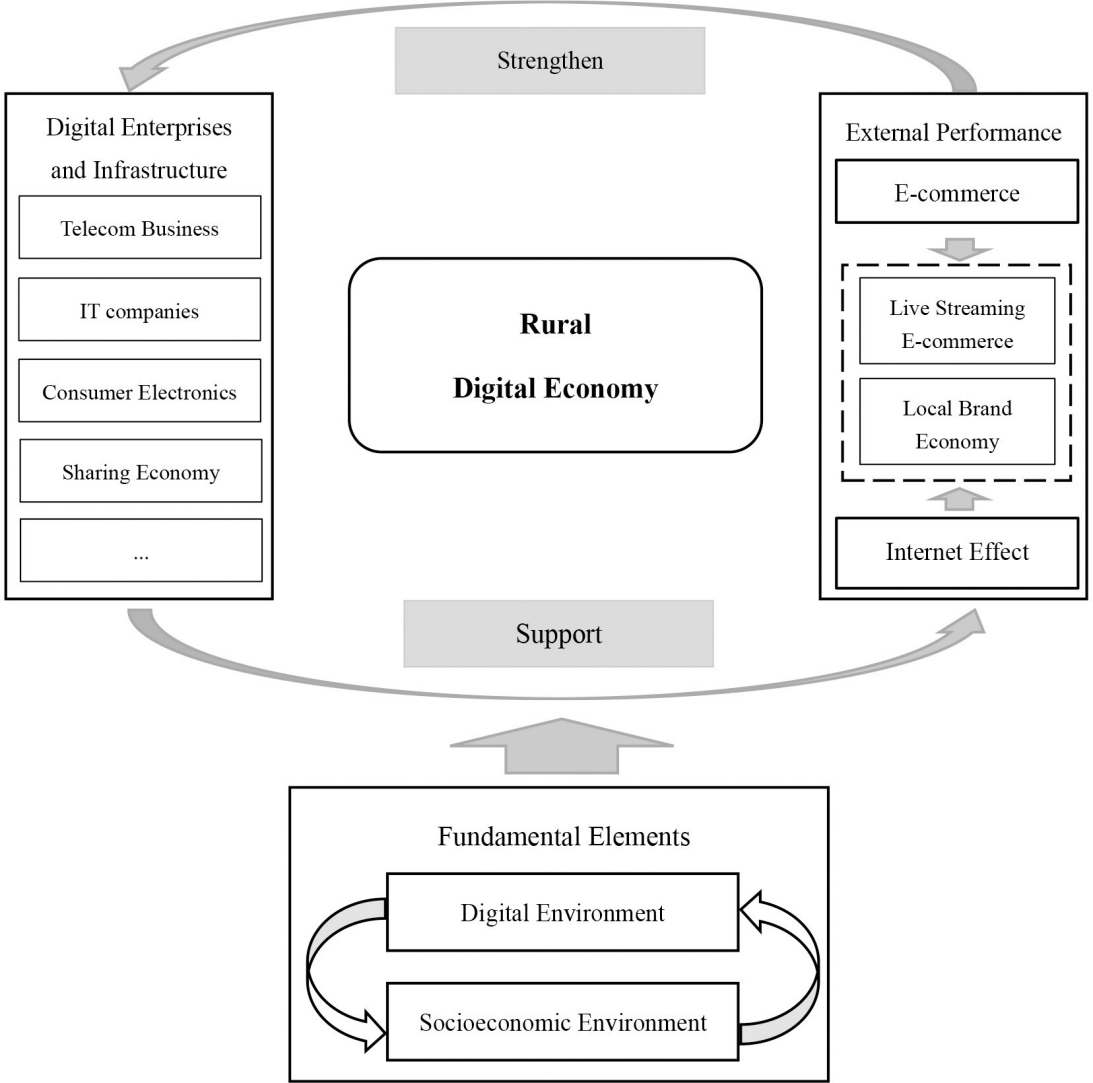
Factors affecting China’s rural digital economy.

We used the entropy weight and spatial differentiation of each indicator to analyze the factors affecting spatial differentiation in rural digital economies. From the calculation results (Table 1), we can see that, in addition to the Internet effect with a weight of 0.484, the important influencing factors were e-commerce level and development vitality, with weights of 0.162 and 0.069, respectively. However, the weights of the industrial environment and digital index were 0.057 and 0.02, respectively. This is mainly attributable to the fact that industrial and digital environments only play a procedural role and lack significant explanatory power. Thus, we offer a comprehensive explanation of the influencing factors by combining the roles of different indicators. Fig 4 shows the results, which we explain in detail below.

(1) Fundamental elements. The digital environment and socioeconomic environment are reflected by the industrial environment and digital index in the evaluation system. They are fundamental elements supporting the development of rural digital economies and are especially important in the initial stage of development. From the spatial pattern, the digital and socioeconomic environments basically determine the distribution pattern of the rural digital economy, although their weights are not high. Compared with the digital environment, socioeconomic aspects, such as GDP, industrial structure, and urbanization level, are more fundamental for digital economy development. A good socioeconomic environment can better support the digital environment, and a better digital environment (e.g., digital village and digital finance construction) can improve the socioeconomic environment.

(2) Digital enterprises and infrastructure. The digital economy is inseparable from the physical support of digital economy enterprises and improved infrastructure construction. Thus, digital vitality is a good indicator for evaluating county-level digital economies, such as the number of telecom business halls, Internet technology companies, digital electronics stores, and shared equipment stations. We found that POIs were densely distributed in urban agglomerations such as the Yangtze River Delta, Beijing–Tianjin–Hebei, Pearl River Delta, Chengdu–Chongqing, and Central Plains, reflecting the vitality of innovation in these regions. These innovation carriers and service centers can not only support the rapid development of regional digital economies but also overcome problems such as a lack of knowledge, technology, and management concepts. Therefore, e-commerce groups can be aided by production, sales, and services from these innovative carriers and service centers. In the future, the digital economy industrial chain and industrial clusters will also be rebuilt to support the real GDP.

(3) External performance. External performance includes e-commerce and the Internet effect. The level of e-commerce development reflects the integration capability of online and offline economies. This stimulates industry vitality and promotes the transformation and upgrading of the economy. It can therefore be an important driving force for digital economy development. The Internet effect can not only enhance the publicity effect of high-quality corporate brands and network delivery experts but also form a characteristic industrial model combined with local characteristics. It has effectively accelerated digitalization in eastern China. High-level counties in eastern China can easily introduce immediate effects and herd effects through the amplification effect of the Internet, whether through the Taobao economy, live-streaming e-commerce, or innovative brands.

## Discussion

**Fig 5:**
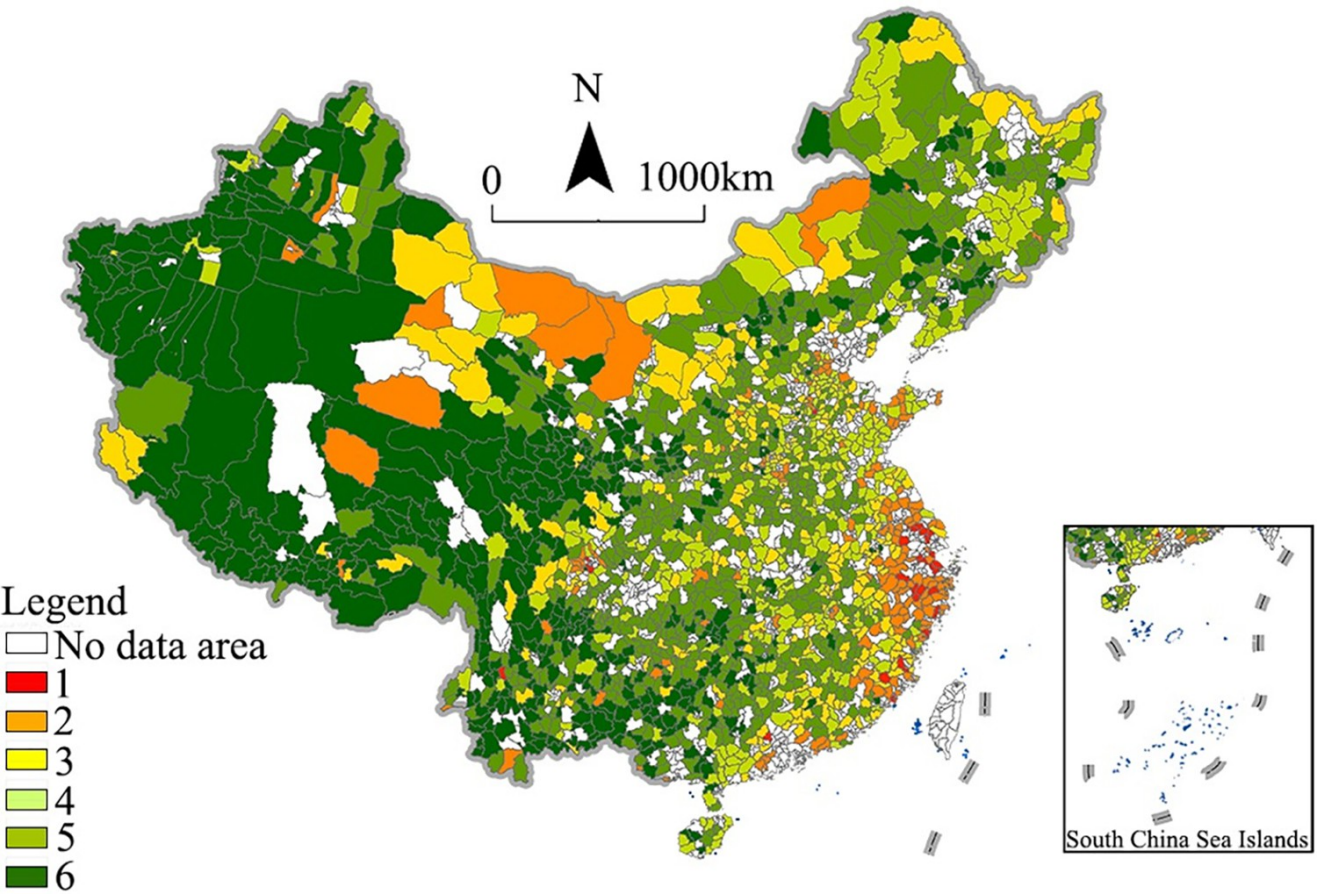
Spatial distribution of rural digital economy types in China.

We found that development vitality, e-commerce level, and Internet effect were the core driving factors of the digital economy. To more deeply investigate the drivers of different types of regions, we used SOFM to classify development types. First proposed by Kohonen [59], SOFM is an unsupervised model that learns only from input data without an external teacher or judgment instruction. It has been widely used in classification problems in geographical research because of its good classification performance and interpretability. The biological basis of SOFM networks is that they learn evolution in a given pattern and extract features or rules from the data. The learning process of SOFM can be found in [60]. Referring to previous studies and considering the number of required classifications, we used three competition layers (2x2, 2x3, and 3x3) and set the number of iterations to 10,000 to train each network. After comparison, we chose a neural network with a 2x3 competition layer and classified the county-level digital economy development types in China. MATLAB R2019a was used for all processes. The classification is explained as follows (Fig 5 and Table 6).

**Table 6:** Explanation of county-level digital economy types in China.

Category	Development vitality	Internet effect	E-commerce level	Development type	Accounts for
**Class I**	0.3203	0.2203	0.3838	E-commerce dominated	1.44%
**Class II**	0.1987	0.1716	0.1845	Balanced high level	8.49%
**Class III**	0.1552	0.0635	0.1119	Vitality + e-commerce	14.04%
**Class IV**	0.1136	0.1642	0.0836	Internet effect dominated	18.05%
**Class V**	0.1022	0.0616	0.0636	Vitality dominated	35.66%
**Class VI**	0.0481	0.0357	0.0431	Low quality	22.32%
**Total average score**	0.1109	0.0862	0.0842		

In Class I and Class II, the counties are mainly distributed in areas with high-level or medium-high-level digital economies. They only accounted for 1.44% and 8.49%, respectively, but were concentrated on the east coast, and only a few were scattered in developed areas in central or western China. Although the regions of Class I and Class II have a similar distribution, their development paths are different. Class I could be called the e-commerce-dominated class; the e-commerce level in these regions is much higher than the development vitality and Internet effect. Compared with Class II, Class I regions have a higher digital economy level with a higher e-commerce level. This further explains the leading role of e-commerce in the digital economy and its significance to the rural economy. For Class II regions, their digital economies might be improved by the surrounding megacities or benefit from their good industrial environments. Therefore, the digital economy and even industrial transformation in Class II regions still need to form a certain leading force in the future.

In Class III, the county-level digital economy is directed by the development vitality and e-commerce level of the area. These counties do not have significant concentrated patterns. However, they typically have medium-level digital economies, which are affected by the trickle-down effect of developed areas or based on good resource endowments and high levels of urbanization. These advantages have laid a good development foundation for these regions. They can therefore construct a solid digital foundation and even develop some micro-digital economic carrier platforms and micro-network technology companies. In this digital application environment, rural residents can conduct e-commerce business activities to a certain degree; thus, this type of county also has a good e-commerce level. However, the low Internet effect limits the digital economy in these counties. Therefore, to improve local digital economies, the government should pay attention to the promotion of characteristic products to increase online attention level.

In Class IV and Class V, the amounts are quite large, accounting for 18.05% and 35.66%, respectively. These are mainly distributed in central and northeastern China with medium or low-medium digital economy levels. Specifically, Class IV is the opposite of Class III, which has low development vitality and low e-commerce levels but a good online attention level. This structure is unsustainable if the counties do not have a good industrial environment. It is important, therefore, to establish better digital infrastructure, digital applications, and e-commercial in the future. Compared with Class IV, Class V regions only have a slightly higher development vitality level and are mainly distributed in medium-low-level areas. This means these counties might only construct fundamental digital infrastructure and primary applications. Although the micro-digital economy is active, these regions should improve the e-commerce level and Internet effect to give full play to the role of micro-digital economy carriers and achieve a high digital economy level.

The counties in Class VI can be considered low-quality areas. The amount and distribution are similar to those of areas with low-level digital economies. These counties are concentrated in southwestern, northwestern, and northeastern China. For these areas, it is more important to provide a solid industrial foundation for developing the digital economy. However, improving the e-commerce level and Internet attention could also become the local path for developing the digital economy after constructing a better digital environment, especially through live-streaming e-commerce and promoting the local brand economy. This is attributable to the fact that these counties usually have a low population density and urbanization level. They might have difficulty developing a large number of digital companies or services.

As a new model, the digital economy is accelerated by informatization and technological modernization. It has become a new driving force for regional growth. According to our results, however, the development of the rural digital economy is still in the initial stage, and unbalanced development is prominent in different provinces and urban agglomeration regions. The main reason is that the digital gap has not been eliminated between different regions [61–65]. Under the influence of the digital gap, developed regions have obtained various advantages in technology, policy, investment, and industry. They have high levels of digitalization and digital industry to stimulate regional vitality and promote industrial transformation and upgrading. By contrast, underdeveloped areas are still in the primary stage of industrialization and informatization and therefore have difficulty accumulating positive growth factors.

However, the Internet effect, industrial vitality, and e-commerce level of the digital economy have become the dominant factors of rural modernization in some underdeveloped regions. Therefore, on the one hand, we should focus on improving the level of digitalization in less developed areas. On the other hand, it is also necessary to focus on improving the Internet effect and e-commerce level and promoting the micro-digital economy to overcome the locking effect of path dependency. Thus, the processes of rural revitalization and coordinated regional development will be accelerated, and even the energy structure and environment will be improved as well.

**Fig 6:**
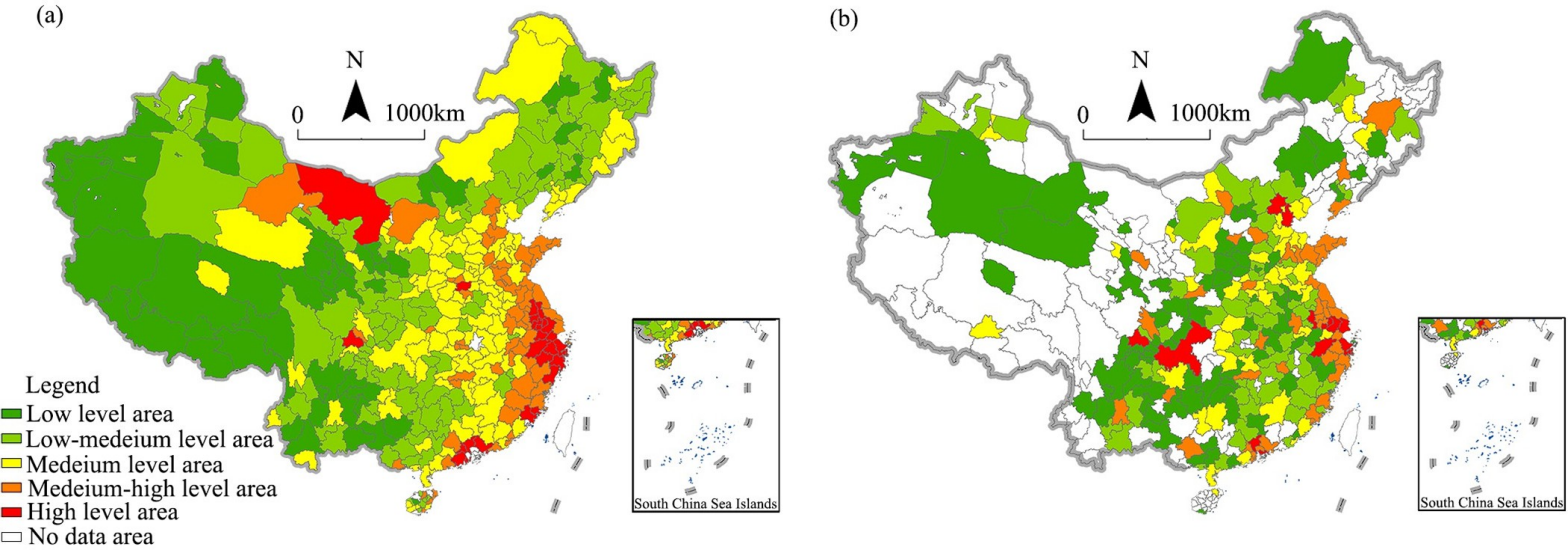
Comparative analysis of the spatial pattern of the digital economy at the city level. (a) Average of county-level digital economy, (b) H3C’s city-level digital economy index.

**Fig 7:**
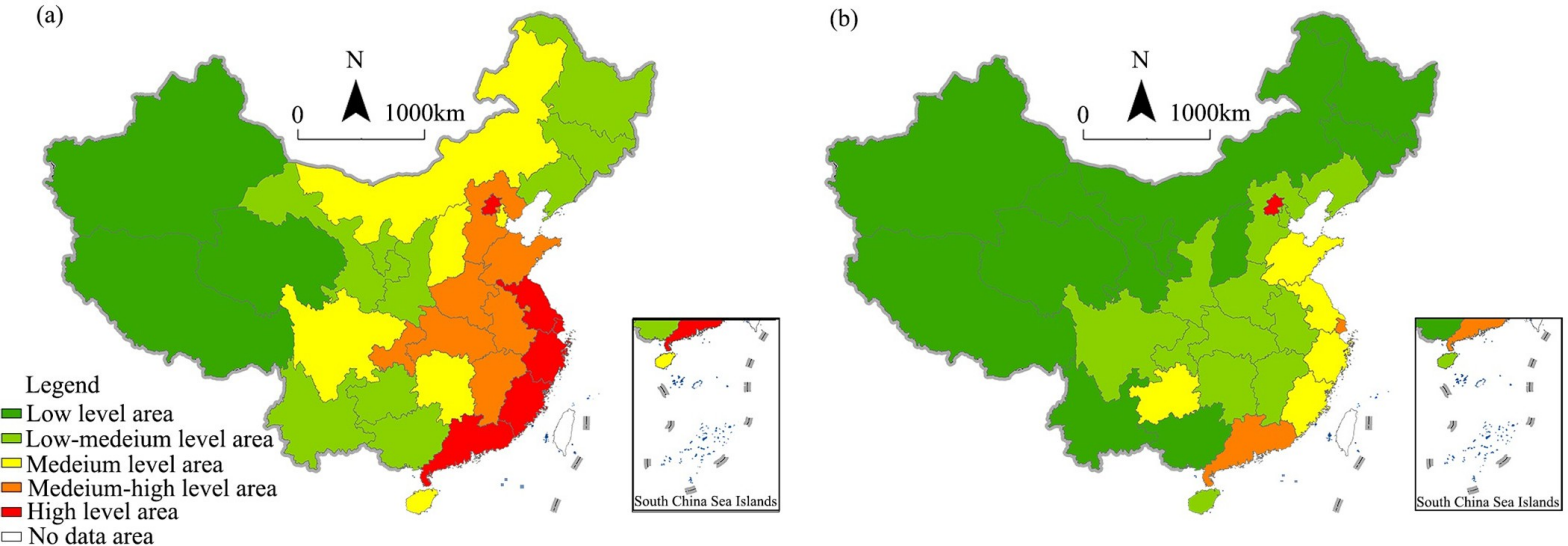
Comparative analysis of the spatial pattern of the digital economy at the province level. (a) Average of county-level digital economy, (b) Wang’s province-level digital economy index.

To further examine the differences between the digital economy at the city level and provincial level, we carried out a comparison based on H3C [33] and Wang et al. [34]. We also used the natural break classification method to divide the scores into five categories; Figs 6 and 7 show the results.

We can see in Fig 6 that the overall spatial pattern is roughly similar, showing the highest level of agglomeration in the Yangtze River Delta Region, some high-level agglomeration in the Pearl River Delta Region, and scattered high-level agglomeration in central and western China. However, we identified not only a continuous belt of high-level areas on the eastern coast but also high-level areas in the border regions with high-level urbanization and nonagricultural economies. The reason could be that the H3C index focuses on evaluating urban digital economies dominated by digital industrial agglomerations and high-level digital applications, while our study focused on rural digitalization processes and basic digital applications.

At the provincial level, in both our research results and Wang’s (Fig 7), the digital economy level shows a gradually decreasing trend from the eastern coast to the western inland area. However, the differences between provinces are smaller, and the internal differences within the Beijing–Tianjin–Hebei and Pearl River Delta regions are larger in Wang’s results. This is also because the Wang’s indicators focus more on urban digital industries. The regional differences in the urban digital economy industries are even more significant. We also found that, although the differences between the provinces in terms of overall digital level are relatively small, the internal difference degree of digital economic levels in Beijing–Tianjin–Hebei and the Pearl River Delta are relatively large. This shows that county-level research can provide more details about the digital economy.

## Conclusion

This study collected different kinds of county-level data and constructed an indicator system to determine the status of China’s rural digital economy. To this end, we combined qualitative and quantitative methods, classic spatial analysis methods, and neural network models. On that basis, we analyzed the geographical characteristics, influencing factors, and development types of the rural digital economy. Based on our theoretical analysis, we provide suggestions for the development of rural digital economies in different regions. The main conclusions are as follows:

(1) From the spatial distribution pattern, the high and medium-high-level areas have formed several agglomeration groups in the eastern coastal area, especially in the Beijing–Tianjin–Hebei region, Yangtze River Delta, and Pearl River Delta. However, their scope and internal connectivity differ significantly. In general, the Yangtze River Delta has the highest rural digital economy level and local ties, especially the counties (or districts) in Zhejiang and Shanghai. Compared with the Beijing–Tianjin–Hebei region and Pearl River Delta, the Yangtze River Delta has a better regional collaborative effect in the rural digital economy and even plays a leading role in China. Therefore, on one hand, the low and low-medium level areas should strengthen their digital infrastructure and applications. On the other hand, in the Beijing-Tianjin-Hebei region and Pearl River Delta, the high and medium-high level areas should export their industrial advantages to the less developed areas around them in order to enhance the overall digital economy level of the entire agglomerations.

(2) From the spatial autocorrelation pattern, the significant HH areas had formed a strip shape from the Beijing–Tianjin region to the Yangtze River Delta and the Pearl River Delta in the coastal region. This is mainly because the degree of economic interaction and infrastructure sharing within these regions is high. The significant LL counties are mainly distributed in western China, especially in the Qinghai–Tibet Plateau and the border areas of northwestern China. In these regions, the geographical environment and industrial basis restrict the rural digital economy. In light of this situation, the eastern coastal region should enhance its support to the western region in terms of digital technology and talent. Consequently, the western region possesses unique potential to elevate the level of its digital economy. It is worth mentioning that leverage the distinctive natural scenery and cultural characteristics of the western region can serve as advantages for developing Taobao economy and live-streaming e-commerce.

(3) From the internal difference pattern, the high-disparity-degree areas are Qinghai and Tibet. In these provinces, the industrial environment varies markedly from county to county. The medium-high disparity degree areas include not only low- and low-medium-level areas such as Xinjiang, Sichuan, and Yunnan but also high-level areas such as Shanghai and Guangdong in eastern China. The medium-disparity-degree areas include not only Zhejiang and Fujian with high-level economies in eastern China but also medium-level areas like Hunan and even underdeveloped areas like Gansu and Inner Mongolia. The low-medium and low-disparity-degree areas are mainly distributed in provinces with low economic and informatization levels in eastern and central China. Developed and less developed provinces should approach internal differences differently. In developed provinces, high-level and medium-high-level counties should enhance their support to other counties in the digital industry and application. In less developed provinces, certain counties should be encouraged to develop their own digital brands, thereby stimulating the digital economy of the entire province.

(4) From the analysis of influencing factors, digital network performance, e-commerce level, and economic vitality were identified as the core factors influencing the rural digital economy. Considering the interplay of these influencing factors, it is crucial to concentrate on two key aspects for the future development of China’s digital economy. Firstly, efforts should be made to improve the level of digitalization in less developed areas. Secondly, emphasis should be placed on enhancing the Internet effect and e-commerce level, as well as promoting the micro-digital economy to overcome the path dependency that can hinder progress. By doing so, the process of rural revitalization and coordinated regional development will be accelerated, and even the energy structure and environment will be improved as well.

## Supporting information

S1 Data (XLSX)
